# Cases of Carditis

**Published:** 1831-04

**Authors:** Charles Mitchell

**Affiliations:** Surgeon.


### CARDITIS.

*Cases of Carditis.* By Charles Mitchell, Surgeon.

The heart, from its importance to the animal economy, as the centre of circulation and organization, requires our most earnest attention, when deranged either in its functions or structure, as they are alike important, not only as impeding the natural action, but deranging the whole vascular system, and considerably endangering life itself. That organic changes are, in most instances, the consequences of inflammation, more or less acute, are, I believe, points scarcely disputed. The primary reduction of carditis, therefore, requires energy and decision, not only to obviate immediate danger, but as a preventive of the secondary effects, which become the most afflicting and irremediable maladies of the human heart.

The cases detailed by Sir D. Dundas are amply sufficient, if such were necessary, to prove the obstinacy and consecutive consequences, nay, even the fatality itself. The subsidence of the inflammatory action in these cases, by means of mercurialism, was an accidental circumstance; but, after a little reflection, it appeared pretty plausible, in consequence of the great advantage which has resulted from its administration in puerperal peritonitis, and likewise independent of the parturient state, more particularly in those cases where the blood does not pourtray the least apparent trace of inflammatory action: although the tenderness is exquisite, accompanied with shrunk features, and attended with sickness, nausea, and vomiting, with a small, weak, and rapid pulse, in such cases, large doses of opium, and the application of leeches to the abdomen, I have found powerful, nay indispensable, auxiliaries.

Mary B., ætat. thirty-five, after supper, at which she ate heartily of animal food, was seized with languor and long-continued faintness, which at length succeeded to a fit of syncope, followed by two hours' collapse of the circulatory system, accompanied with great oppression and difficult respiration. The reaction ensued without the least perceptible rigor, but attended with excruciating pain, oppression, difficult and laborious breathing, tightness, inability to lie down, with much marked anxiety, and a great sensation, as if the heart would burst from distention. In this almost insupportable state of suffering, I saw her, and extracted blood to syncope, (which was cupped and buffy,) with considerable, although temporary, relief. I was again called to her by six o'clock the following morning, when I found the sufferings greater than they were the preceding evening, attended with restlessness, and the feeling of distention aggravated, with repeated exclamations, "Oh, sir! my heart will burst!" which required a few efforts, in consequence of the continued laborious respiration, accompanied with agonising pain, cough, oppression, a cordy tightness, with pulsatory influence over the whole chest, from its force and frequency. I again bled her to syncope, and ordered, besides the administration of a purgative, the following powder, to be taken every two hours: ℞. Pulv. Digital. gr. i.; P. Gum Camphor. gr. i.; Hydrarg. Submur. gr. i. M. ft. p.

I called again in the course of five hours, and found the blood cupped and buffy; but the symptoms, from being somewhat remedied, had again resumed their aggravated appearance, with a renewal of the acclamations, although she felt, upon the whole, better, suffering less from the pain, oppression, tightness, or difficulty in moving. Rep. pulv.

In the evening, I was again requested to see her, when she complained of the pain being most excruciating, with a feeling as if the heart would burst, and a renewal of all the aggravated symptoms. She was immediately bled to syncope, (cupped and buffy, with the coagulated lymph upon its surface,) and took three grains of calomel and one of opium.

I was again called to her by seven o'clock the next morning, when I found her occasionally insensible, accompanied by violent writhings of the body, contractions of the fingers, rolling and squinting of the eyes, with a quick, irregular, and small pulse. In two or three minutes, this state subsided; she became motionless for awhile, with quick and broken breathing; lips of a palish hue. The eyelids quivered a little prior to their being properly opened, (when she made a snatch at the region of the heart, as if she attempted to pluck it out.) When she became more rational, she frequently exclaimed, her heart would burst, and that she suffered likewise from severe pain of the back, extending down the arms, attended with numbness of the fingers. A convulsive fit of coughing occasionally seized her, attended with tremors of the limbs, followed by heaving at the chest, with a relapse to the state of convulsive action and insensibility.

In the course of one hour and a half, these fits, in some degree, abated; but, in consequence of the excruciating pain, difficult respiration, cough, tightness, anxiety, and feeling of distention, being of an urgent nature, and, at the same time, from the frequent recurrence of uneasy sensations, the entire subsidence of the paroxysms was prevented. I, however, repeated the bleeding to syncope, (blood cupped and buffy.)

In the evening, the mouth was severely affected, but she was much improved. The powders were continued, without the calomel.

In the morning, twelve ounces of blood extracted, because the cough and oppression still continued. I detracted sixteen ounces more blood; and, finding her very little improved in the evening, twelve more were withdrawn, in order to allay the existing action. In the morning, the breath had acquired slightly the mercurial fœtor, accompanied with tenderness of the gums. The aggravated symptoms were in a great measure subdued.

The powders were now only administered every four hours, but were altogether omitted in the evening, from the constitution being severely influenced by the mercury. The cough still continued troublesome; the pulse quick, with a very uneasy sensation about the region of the heart. She was cupped to eight ounces; took two grains of opium at bedtime, which procured a most refreshing sleep of four hours, with the subsidence of the irritative action in a great degree. She likewise took the following every four hours: ℞. Mucil. Acac. ʒss.; Tinct. Scillæ ♏xv.; Tinct. Digital. ♏xv.; Mist. Camph. ℥iss. M.

The uneasy sensation subsided, the cough became less troublesome, and she rapidly recovered.

Mrs. G., ætat. forty, a strong plethoric woman, was seized with occasional faintness for two days, when it approached almost to a state of syncope, followed by languor, and sometimes pain of a spasmodic nature about the region of the heart, attended with short breathing, small and contracted pulse. Stimulants (ammonia and ether) were administered, but, from the approach of severe rigors, their use was wisely dispensed with by the patient. The rigors were followed by severe pain, aggravated upon the slightest movement or extension of the body, which consequently confined her in a sitting posture, with the body bent forwards. The breathing was now short and difficult, attended with cough, oppression, and tightness, with a full, firm, and quick pulse. I bled her to syncope, and administered the following powder every two hours: ℞. Antimon. Tart. gr. $\frac{1}{4}$; Pulv. Opii gr. $\frac{1}{2}$; Submur. Hydr. gr. i. M. ft. pulv.

When I called in the morning, I found a mass of coagulated lymph occupying the cup cavity of the inflamed blood, only adhering to its margins. She felt much annoyed with uneasy sensations about the region of the heart, attended with considerable increased action of the heart and arteries. The blood was buffy, but not cupped.

Under the use of the powders, without the calomel, the woman rapidly recovered.

Mrs. A., ætat. thirty, about eight months pregnant, had, for eight or ten days, felt very unwell, but now much worse, in consequence of repeated and severe rigors, accompanied with pain which came on suddenly, palpitations, occasional syncope, inability to lie down, great oppression, and a feeling of distention as if the heart would burst; pulse small and constringed. In this state I was called to her one morning about two o'clock. I immediately bled her to syncope, after which she expressed herself much relieved.

I was again called to her betwixt four and five in the morning, and found her in a similar state to that in which I found her the preceding part of the morning. I again bled her to syncope, after which she experienced great relief, and could lie down in bed, very little incommoded. These bleedings displayed very little trace of inflammatory action. An anodyne draught was administered.

Uneasy sensations, and occasionally palpitations, occurred during the remaining part of gestation, but finally subsided after parturition.

*Lamb's Conduit street; February* 11*th*, 1831.

