# Errata in the Last Number

**Published:** 1831-05

**Authors:** 


### *Errata in the last Number.*

P. 294, 16 lines from the bottom, for "snack" read "snatch."

In Mr. Mitchell's paper on Carditis, several transpositions occurred, in consequence of the paging of the manuscript being altogether omitted. To the words "with a renewal of the acclamations," p. 294, ought to have succeeded "I extracted sixteen ounces of blood," &c. p. 295, down to the conclusion of the case. In the second Case, from "the increased action of the heart and arteries," p. 296, should have followed "although she felt," &c. p. 294, to the third line at top of p. 295, and afterwards, in continuation, from "attended with considerable increased action," p. 296, to the conclusion of the case.

